# Modeling electrochemical properties of LiMn$_{1-x}$Co$_{x}$BO$_3$ for cathode materials in lithium-ion rechargeable batteries

**DOI:** 10.1038/s41598-021-90317-0

**Published:** 2021-06-04

**Authors:** Sérgio Leonardo Nhapulo, Jailton Souza de Almeida

**Affiliations:** 1 Instituto de Física, Universidade Federal da Bahia, Campus Universitario de Ondina, Salvador, Bahia 40210-340 Brazil; 2 Faculdade de Ciências Exatas e Tecnológicas, Universidade Púnguè, Estrada Nacional Nº 6, Caixa Postal 323, Chimoio, Manica Mozambique

**Keywords:** Batteries, Electronic properties and materials

## Abstract

In this work, we report first-principle calculations of the electrochemical properties of lithitated and delithiated LiMn$_{1-x}$Co$_{x}$BO$_3$ ($x = 0$, 0.25, 0.5, 0.75, 1) crystals based on the density functional theory (DFT) with the generalized gradient approximation (GGA) and also considering the on-site Coulomb interaction, the so-called Hubbard correction. We found that the top of the valence band and the bottom of the conduction band of these crystals are mainly formed by the hybridization of the 3d orbitals of mixed Mn$_{1-x}$Co$_{x}$ ions and oxygen 2p orbitals. We observed a band gap narrowing with an increase of cobalt concentration and that the Hubbard correction implies a better theoretical description of their electronic structures. When considering the delithiated materials, our calculations show a metallic behavior for intermediate cobalt concentrations ($x = 0.25$, 0.5, 0.75), which is a good quality for cathodic materials, as it improves the battery discharge process. We also obtained high (4.14 V vs. Li$^+$/Li$^0$ and 4.16 V vs. Li$^+$/Li$^0$) open circuit voltage (OCV) values at cobalt concentrations of $x = 0.5$ and 0.75, where we believe that if these high OCV values are accompanied by a high charge storage capacity, these compounds can become promising and useful cathode materials. Finally, our results are in accordance with previous calculations and also with experimental results.

## Introduction

Over the last four decades, the evolution of portable electronic devices as well as electric vehicles and hybrid electric vehicles has created a greater demand for energy storage systems and, as a consequence, storage systems with higher capacity, or with reduced weight and size for adequate capacity, have become even more necessary^1^. In addition, conventional rechargeable batteries such as nickel-cadmium, lead-acid and nickel-metal hydride batteries, which were in mass use and development at the time, imposed limitations in terms of size and weight reduction, making it necessary to implement new, smaller and lighter rechargeable battery technology^2–5^. On the other hand, recent studies on lithium-ion rechargeable battery cathode materials have mainly focused on the polyanionic structure^6^ because they exhibit many desirable properties such as high energy density. It is worth mentioning that there are already excellent published articles that comprehensively analyze the polyanionic cathodes used in lithium ion batteries^7–11^.

Batteries and other types of electrochemical devices are basically regulated by three main physical processes: charge separation, transport of charged species and charge recombination^12^. The fundamental concept behind the storage of electrochemical energy system is the reciprocity between the conversion of the chemical energy stored in the fuel connections into electrical energy and the expenditure of electric energy to synthesize chemicals or fuels operating in the reverse direction^12,13^. A more detailed explanation of the principle of operation of batteries can be found elsewhere^14^.

To date, the majority of the research on battery has been based on rechargeable lithium-ion batteries due to the greater electropositibility of Li (Li$^+$/Li$^0$ whose redox potential $x = -3.04$ V vs. standard hydrogen electrode (SHE)) and high energy density^4,5,12,15^. In lithium-ion batteries, the extraction of the Li ion at the cathode (whose working potential is higher than 2 V vs. Li$^+$/Li$^0$) is observed during the battery charging process, and intercalated at the graphite anode (whose working potential is lower than 3 V vs. Li$^+$/Li$^0$)^3,12,16^ and, as a result of this electrochemical process, the free electrons obtained from the chemical reaction, Li = Li$^+$ + e$^-$, move through the external circuit carrying out work^13^. During the charging process, lithium-ion is extracted from the positive electrode, whose working potential is higher than 2 V vs. Li$^+$/Li$^0$ and, intercalated into the graphite anode, whose working potential is lower than 3 V vs. Li$^+$/Li$^0$^3,12,16^ and, as a result of this electrochemical process, the free electrons obtained from the chemical reaction, Li = Li$^+$ + e$^-$, move through the external circuit carrying out work^13^. Consequently, these extremely attractive properties led to lithium-based battery systems gaining more attention from researchers and investors, and as a result, Sony Corporation stood out as the first company to bring ion-ion batteries to market in 1991^12,17^.

However, one of the main drawbacks of Li-ion batteries is in the materials for the cathode because of their limited energy densities^2^. In order to allow good reversibility and good life cycles, lithium-ion batteries need to possess a cathode that during their operations present the smallest volumetric change possible. Due to their low cost, high safety and because they are benign to the environment, olivine type phosphates were strongly considered as the polyanions that could be used as cathodes for lithium-ion batteries. Unfortunately, their specific capacity is limited to 170 mAh/g and consequently their energy density is also limited to 586 Wh/kg with moderate operating voltage (3.45 V vs. Li$^+$/Li$^0$)^18^ which hinders the batteries’ performance.

The research of new cathodic materials with high capacity, good stability, and high safety is important to improve the performance of Li-ion batteries. Recently, borate materials containing transition metal (TM) atoms in their composition like LiMBO$_3$ (M = Fe, Mn, Co) have been pointed out as good alternatives when compared to the phosphates since they have high specific capacity (above 210 mAh/g) and also keep the advantage of safety^19–21^. However, the redox potentials of couples Fe$^{2+}$/Fe$^{3+}$ (3 V vs. Li/Li$^+$)^22^ and Mn$^{2+}$/Mn$^{3+}$ (3.7 V vs. Li/Li$^+$)^23^ are relatively low which limits the energy density of the LiFeBO$_3$ and LiMnBO$_3$ compounds. On the contrary, it is found that LiCoBO$_3$ enables to increase the energy density giving that couple of Co$^{2+}$/Co$^{3+}$ has a higher redox potential (4 V vs. Li/Li$^+$)^24^ than couples Fe$^{2+}$/Fe$^{3+}$ and Mn$^{2+}$/Mn$^{3+}$. Unfortunately, the disadvantage of the LiCoBO$_3$ compound to be considered as a cathodic material is its rather low experimental reversible capacity when compared to LiFeBO$_3$ and LiMnBO$_3$^25^.

Bearing all these facts in mind, in this work we report first-principle calculations of electrochemical properties of the LiMn$_{1-x}$Co$_{x}$BO$_3$ ($x = 0$, 0.25, 0.5, 0.75, 1) in order to improve the understanding of properties of lithium borate-based materials to help in the design of new materials that can be satisfactory with respect to energy density, specific capacity and stability during battery charging and discharging cycles. On the other hand, although iron improves conductivity, its absence makes the compound less sensitive to surface air poisoning^20,26,27^, which, in a way, can contribute to increasing battery performance.

## Computational details

Calculations of electrochemical properties of LiMn$_{1-x}$Co$_x$BO$_3$ were performed by solving the electronic-structure problem within density functional theory (DFT)^16^ using the *Vienna ab initio simulation package* (VASP) software^28^. The spin-polarized calculations were carried out employing the projected augmented wave (PAW) method together with the generalized gradient approximation (GGA) with Perdew, Burke, and Ernzerhof (PBE) parametrization^16,29^ for the exchange-correlation functional. We have also considered the localization of 3d electrons of the transition metal ions in these materials by including the Hubbard term in the Kohn–Sham scheme following the approach by Dudarev et al.^30^. Following Seo et al.^21^, we have used the Hubbard U value of 4.5 eV and 5.7 eV for the 3d orbitals of Mn and Co atoms, respectively. Such values were shown by previous investigations to be suitable values for LiFe$_{1-x}$M$_x$BO$_3$ (M = Mn, Co, and Ni) crystals^31^. The PAW potentials with valence states 1s for Li atom, 2s and 2p for B and O atoms, and 3d and 4s for Mn and Co atoms were used. A basis set up to a kinetic energy cutoff of 500 eV have been used and the integration over the Brillouin zone was performed using a $3\times 3\times 3$ k-points grid obtained with Monkhorst–Pack method. All crystal structures were set up and analyzed using VESTA^32^ software. To allow the desired Mn/Co content in the crystal structure, we built $2\times 1\times 1$ supercells^33^ starting from the experimental lattice constants of the monoclinic crystal structure with C2/c space group^20^. All the calculations have been done until the Hellmann–Feynman forces become smaller than 10$^{-3}$ eV/Å and the total energies converged to below 10$^{-4}$ eV.

The average open circuit voltage (OCV) for LiMn$_{1-x}$Co$_x$BO$_3$ crystals was calculated as

$$\begin{aligned} OCV = \frac{-E({{\mathrm {LiMn}}_{1-{\mathrm {x}}}{\mathrm {Co}}_{\mathrm {x}}{\mathrm {BO}}_3})+E({{\mathrm {Mn}}_{1-{\mathrm {x}}}{\mathrm {Co}}_{\mathrm {x}}{\mathrm {BO}}_3})+yE({{\mathrm {Li}}})}{ye} \end{aligned}$$

where *x* is the concentration of cobalt, E(LiMn$_{1-x}$Co$_x$BO$_3$) and E(Mn$_{1-x}$Co$_x$BO$_3$) are the total energies of fully lithiated and delithiated materials, respectively. E(Li) is the total energy per atom of the lithium metal in the bbc structure, *y* is the number of lithium atoms in the cell and, finally *e* is the electronic charge^16^. Note that a lithium ion battery is considered commercially viable when OCV $\ge$ 4 V^34^.

## Results and discussion

In this section we discuss our results for the crystal structures, the electronic properties by means of density of states (DOS) using both GGA and GGA+U approximations as well as the open circuit voltage (OCV) for these borates.

### Crystal structure

The LiMn$_{1-x}$Co$_x$BO$_3$ materials adopt monoclinic-like crystal structure (space group: C2/c) as synthesized and published in Refs.^20,25,35–37^. In this structure, the transition metals (TM) ions Mn and Co occupy the center of the trigonal bipyramids composed by five oxygens ((MnO$_5$) or (CoO$_5$)) and in turn, the Li are embedded in a tetrahedron with four oxygen atoms around them (LiO$_4$) so that these two different polyhedras (MnO$_5$)/(CoO$_5$) and (LiO$_4$) are connected to each other via corner and edge sharing which are condensed to form a polyhedral chain and, finally, the two polyhedral chains are further interconnected through trigonal planar BO$_3$ units in which the B is located at the center of the planar triangle.

**Figure 1:**
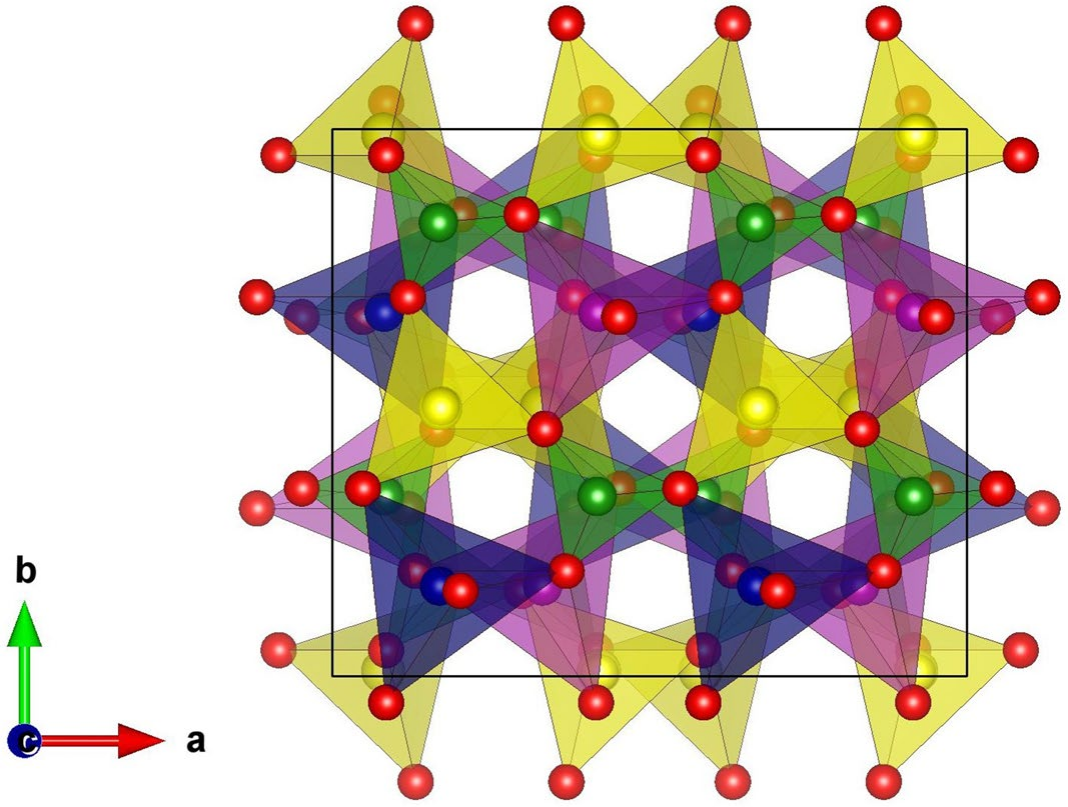
Crystal structure of LiMn$_{1-x}$Co$_x$BO$_3$ materials at $x = 0.5$, where the yellow color represents Li, blue Mn, lilac Co, green B and red O atoms, respectively. The figure was built up using VESTA software.

In Fig. 1, we show the schematic crystal structure of LiMn$_{1-x}$Co$_x$BO$_3$ materials for $x = 0.5$ with eight formula units that possess C2/c space group and monoclinic unit cell. Since the mixed compounds have crystal structures similar to the structural type of the LiMBO$_3$ (M = Mn, Fe, Co), to model their properties is necessary to replace some Mn atoms by Co atoms to achieve the desired concentration.

Table 1 presents our calculated results for the lattice parameters after the supercell structural relaxation procedure and compared them to the experimental values obtained from Ref.^20^. As one can see, the calculated and experimental results are in good agreement having only a few percents of deviation.

**Table 1:** Lattice parameters and angle of each material are presented. The numerator contains the values of our calculations, while the denominator contains experimental values published in Ref.^20^. Changes in the lattice parameters ($\Delta a$, $\Delta b$, $\Delta c$) and changes in the angles ($\Delta \beta$) between the experimental and calculated values are presented.

	*a* (Å)	*b* (Å)	*c*(Å)	$\beta (^{\circ })$	$\Delta a(\%)$	$\Delta b(\%)$	$\Delta c(\%)$	$\Delta \beta (\%)$
LiMnBO$_3$	5.199/5.207	8.973/8.976	10.358/10.381	91.82/91.83	0.15	0.03	0.22	0.01
LiMn$_{0.75}$Co$_{0.25}$BO$_3$	5.186/5.186	8.941/8.941	10.321/10.321	91.69/91.69	0.00	0.00	0.00	0.00
LiMn$_{0.5}$Co$_{0.5}$BO$_3$	5.170/5.170	8.918/8.918	10.256/10.260	91.59/91.59	0.00	0.00	0.04	0.00
LiMn$_{0.25}$Co$_{0.75}$BO$_3$	5.185/5.152	8.940/8.888	10.316/10.194	91.69/91.44	0.64	0.59	1.20	0.27
LiCoBO$_3$	5.134/5.131	8.853/8.855	10.104/10.120	91.38/91.32	0.06	0.02	0.16	0.07

### Electrochemical properties

The electrochemical properties of fully lithiated ($y = 1$) and delitiated ($y = 0$) Li$_{y}$Mn$_{1-x}$Co$_{x}$BO$_3$ ($x = 0$, 0.25, 0.5, 0.75, 1) crystals were studied by means of the density of states (DOS) and the open circuit voltage (OCV) considering the GGA and GGA+U approximations. First we address the results for GGA approximation and thereafter we show the influence of the Hubbard U term on the properties of these crystals.

#### Density of states

**Figure 2:**
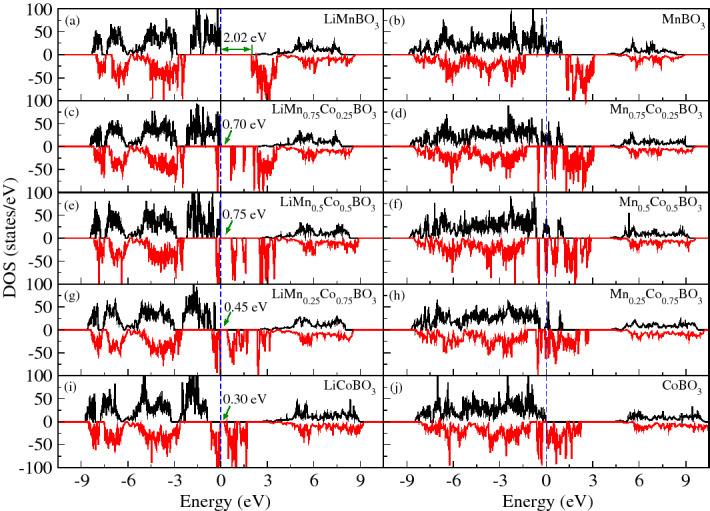
Density of states of fully lithiated ($y = 1$; left panel) and delitiated ($y = 0$; right panel) Li$_{y}$Mn$_{1-x}$Co$_{x}$BO$_3$ crystals calculated using GGA. The Fermi level is indicated by a vertical dashed line.

In Fig. 2a,c,e), (left panel; top-down) we show the influence of increasing cobalt concentration on the electronic structure of these materials. As one can see, the top of the valence band is basically filled with spin-up electrons while the unoccupied states at the bottom of the conduction band are only allowed for spin-down electrons. However, when increasing even further (75% and 100%) Co concentration, Fig. 2g,i, we notice that the highest occupied band as well as the lowest unoccupied band are allowed for spin-down electrons and completely forbidden for spin-up electrons. For LiMnBO$_3$, our calculations show that the upmost valence band, which is filled with spin-up electrons, is made up of a hybridization between manganese 3d and oxygen 2p states, while the spin-down lowest conduction band is mainly formed by manganese 3d states.

When we substitute 25% and 50% of manganese by cobalt at LiMnBO$_3$ crystals, our calculations reveal that the highest occupied band is mostly filled with Mn spin-up electrons on 3d states while the lowest unoccupied spin-down band is formed by Co 3d states. In the cases of 75% and 100%, we note that cobalt spin-down electrons on 3d-states dominates the formation of the valence and conduction bands around the Fermi level.

According to our calculations, when the materials are delithiated (*y* = 0), they become metals for all Co concentrations considered, Fig. 2b,d,f,h,j, (rigth panel; top-down) thus showing good electronic conductivity during battery discharge. For MnBO$_3$, in Fig. 2b, the valence and conduction bands around the Fermi level are mainly composed by Mn 3d and O 2p states, respectively. However, for 25% and 50% of Co concentrations, the top of the spin-down valence band is mostly formed by cobalt on 3d-states and a relatively small amount of manganese 3d-states and oxygen 2p-states. The bottom of the spin-up conduction band is a mixture of manganese of 3d-states and oxygen 2p-states. When considering 75% and 100% Co concentrations in the delithiated materials, our calculations also reveal the predominance of the 3d-states cobalt for the spin-down band around the Fermi level. This is probably due to the fact that the Co$^{2+}$/Co$^{3+}$ couple has a greater redox potential than Mn$^{2+}$/Mn$^{3+}$ couple.

Basically, the situation is as follows: as we gradually increase the concentration of Co, we notice the formation of a spin-down band and the gradual disappearance of the filled spin-up band close to the Fermi level. We also see a bandgap narrowing from 2.02 to 0.30 eV when we increase the Co concentration, the absence of Li-2s states close to the Fermi level is an indication that lithium atom is fully ionized and that the hybridization between Mn (or Co) 3d states and the oxygen 2p states to indicates some degree of covalent interaction. Conclusions similar to these can be drawn from other battery cathode materials^38–42^.

#### Density of states with Hubbard correction

The density of states of fully lithiated and delithiated Li$_{y}$Mn$_{1-x}$Co$_{x}$BO$_3$($x = 0$, 0.25, 0.5, 0.75, 1) materials was also calculated using GGA+U with the following Hubbard U values applied on 3d states of TM; U$_d$(Mn)= 4.5 eV and U$_d$(Co)= 5.7 eV.

**Figure 3:**
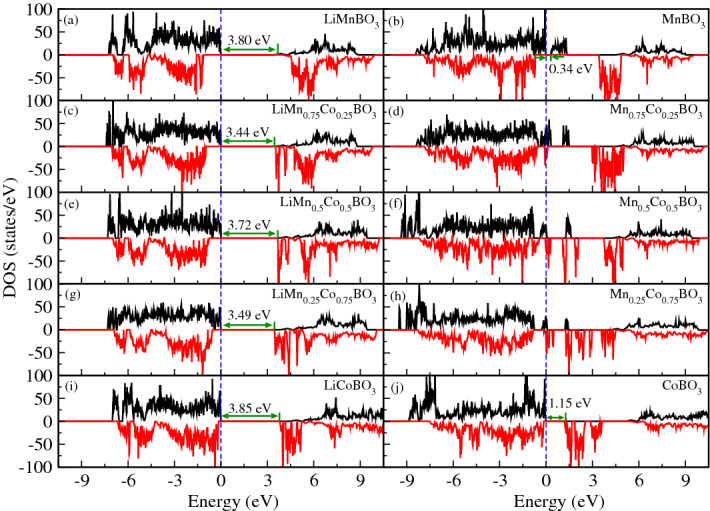
Density of states of fully lithiated ($y = 1$; left panel) and delitiated ($y = 0$; right panel) Li$_{y}$Mn$_{1-x}$Co$_{x}$BO$_3$ crystals calculated using GGA$+$U where U$_d$(Mn)= 4.5 eV and U$_d$(Co)= 5.7 eV. The Fermi level is indicated by a vertical dashed line.

The Fig. 3a shows that both the top of the valence band and bottom of the conduction band are allowed for spin-up electrons and completely forbidden for spin-down electrons. The same behavior is observed when the material is delithiated as shown in Fig. 3b. We notice that the energy bandgap of the lithiated material is significantly increased by the effect of the Hubbard correction and there is a small gap opening for the delithiated case. Such results are consistent with the theoretical study for pure unmixed borate materials presented by Seo et al.^21,38^ and similar bandgap widening has been previously observed in other calculations for olivine phosphates^43^. Conversely, in the other Co concentrations, as shown in Fig. 3c,e,g,i, (left panel; top-down) the highest occupied bands are filled with spin-up electrons and the lowest unoccupied bands are only allowed for the down-spin electrons. The gradual increase of Co concentration in place of Mn causes the spin-down conduction bands to move from right to left while the spin-down valence bands move in the opposite direction approaching the Fermi-level. It is worth noting that, when we add 25% of cobalt in LiMnBO$_3$ material, the filled spin-up valence band is mainly formed by manganese 3d-orbitals and oxygen 2p-orbitals while the bottom of the spin-down conduction band is a mixture of cobalt 3d states and boron 2p states.

In general, the calculations for these materials show that the conduction and valence bands, close to the Fermi level, are mainly composed of 3d-orbitals of Mn$_{1-x}$Co$_{x}$ ($x = 0$, 0.25, 0.5, 0.75, 1) and oxygen 2p-orbitals, also that there is a very small contribution of boron 2p-orbitals.

The delithiated materials Mn$_{0.75}$Co$_{0.25}$BO$_3$, Mn$_{0.5}$Co$_{0.5}$BO$_3$, and Mn$_{0.25}$Co$_{0.75}$BO$_3$, Fig. 3d,f,h), (right panel; top-down) exhibit the desired metallic behaviour for application as battery’s cathode. When considering MnBO$_3$ and CoBO$_3$ (Fig. 3b,j, respectively), however, our GGA$+$U calculations show that they behave like semiconductors with a small gap which can decrease the electronic conductivity during the battery discharge. Nonetheless, it is expected that the band gaps for the delithiated crystals do not play a significant role in the electronic conductivity of borates similar to the way that band gaps do not play a significant role in the electronic conductivity of other insulating intercalation materials such as olivine phosphates. Additionally, the band gaps problem in the Mn$_{1-x}$Co$_{x}$BO$_3$ compounds could be overcome with a temperature rise as pointed out before^21,43^.

#### Open circuit voltage

The open circuit voltage (OCV) is an important characteristic parameter of lithium-ion batteries that is often used to analyze changes in electronic energy in electrode materials, to estimate battery charge status (SOC) and to manage the battery pack^44^. In our calculations, we found that all OCV values calculated using the GGA approximation are about 26 % smaller than the OCV values obtained from GGA$+$U at the same concentration. This result is somewhat expected because the Hubbard correction can better describe the localized 3d states of the TM in strongly correlated systems, such as in these oxides. It has also been found that incomplete cancellation of the self-interaction of the GGA approximation tends to underestimate OCV values when compared to GGA$+$U in olivine compounds^45–47^.

**Figure 4:**
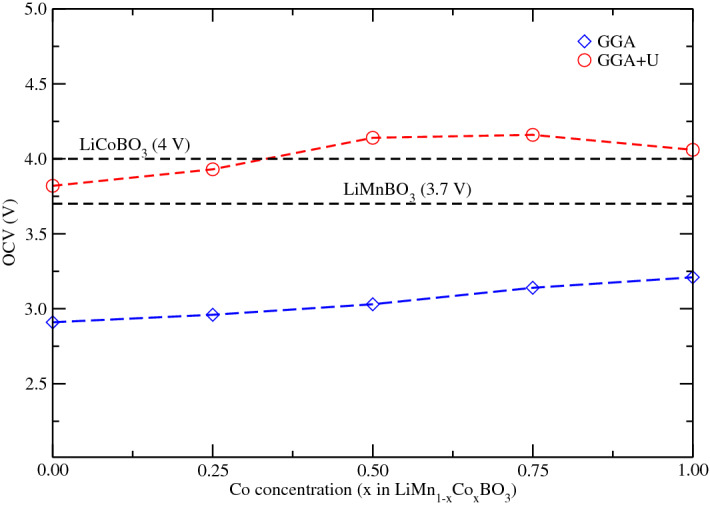
Experimental values (horizontal dashed lines in black)^23,48^ and calculated OCV values versus cobalt concentration for the borate compounds LiMn$_{1-x}$Co$_{x}$BO$_3$ using GGA and GGA+U approximations. We used U$_d$(Mn) = 4.5 eV and U$_d$(Co) = 5.7 eV.

Our OCV results for LiMnBO$_3$ and LiCoBO$_3$ using GGA$+$U are also in good agreement with the experimental results. Figure 4 shows that OCV increases with cobalt concentration and its maximum value is reached at 75% of cobalt in the material using GGA+U. For LiMn$_{1-x}$Co$_{x}$BO$_3$ compounds, we clearly observe that the average OCV values (4.14 and 4.16 V) for 50% and 75% of Co are larger than the values calculated for pure materials which can be associated to the experimental observation of two oxidation peaks at 3.5 and 4.2 V showing that both Mn$^{2+}$/Mn$^{3+}$ and Co$^{2+}$/Co$^{3+}$ couples are active in these materials^20^. Hence, our GGA+U calculations show that LiMn$_{0.5}$Co$_{0.5}$BO$_3$ and LiMn$_{0.25}$Co$_{0.75}$BO$_3$ crystals can become promising alternative cathodes because their OCV values exceeds the calculated values for LiMnBO$_3$ and LiCoBO$_3$.

## Conclusion

The electrochemical properties of lithiated and delithiated LiMn$_{1-x}$Co$_{x}$BO$_3$ ($x = 0$, 0.25, 0.5, 0.75, 1) crystals were theoretically investigated using DFT in the GGA and GGA$+$U approximations. Our calculations show that the valence and conduction bands, close to the Fermi level, are mainly composed by the hibridization of Mn$_{1-x}$Co$_{x}$ 3d-orbitals and oxygen 2p-orbitals. We observed a band gap narrowing by increasing cobalt concentration. Additionally, the electronic properties were corrected by the consideration of the Hubbard term which improves the theoretical description of the localized 3d orbitals of TM atoms. We note that upon delithiation, the materials with the transition metal mixture Mn$_{1-x}$Co$_{x}$ ($x = 0.25$, 0.5, 0.75) behave like metals which favors the electronic conductivity during the battery discharge process. We also observed that in the concentrations corresponding to $x = 0.5$ and 0.75, the crystals show good OCV results since their values are relatively higher than the values of the other concentrations, remembering that a lithium ion battery is considered commercially viable when the OCV value is greater or equal to 4 V. Finally, our results are in good agreement with previous calculations and experimental results for $x = 0$ and $x = 1$. For the others cobalt concentrations, we also found good agreement with experimental findings.
